# The Leprosy Problem and Its Bearing on Tuberculosis

**Published:** 1924-01

**Authors:** Leonard Rogers


### THE LEPROSY PROBLEM AND ITS BEARING ON TUBERCULOSIS.

### AN ABSTRACT OF A POSTGRADUATE LECTURE AT THE UNIVERSITY OF BRISTOL.

BY

SIR LEONARD ROGERS, C.I.E., M.D., F.R.C.P., F.R.C.S., F.R.S., I.M.S.Ret.

---

THE ancient problem of leprosy has entered on a new era as the result of recent advances in its treatment, which is not without some bearing on the still more important subject of tuberculosis, so some account of the present position may be of interest.

*Communicability.*—It is now generally recognised that leprosy is an infectious disease, although it is usually necessary to live for some time in close association with a leper before contracting the disease, which is probably less infectious than tubercle. I recently analysed 700 cases I

collected from the literature of the past five decades, in which the probable source of infection was traced,[1] and concluded that in at least 70 per cent. it was derived while living in the same house, and in not less than 30 per cent. through sleeping in the same bed with a leper ; 95 per cent. of the infective cases having been of the nodular type and only 5 per cent. anæsthetic cases, which are far less infective owing to the lepra bacilli being confined to the nerve trunks and rarely escaping from the body. I also recorded strong evidence in favour of the usually accepted view that the infective organism enters by inoculation through slight abrasions of the skin. For example, two doctors have wounded their fingers while operating on lepers, and in both cases the disease began in the wounded finger. Dr. E. Muir,[2] Leprosy Research Worker in the Calcutta School of Tropical Medicine, found the earliest lesions to be located principally on exposed extensor surfaces, while the high incidence of the disease in damp hot countries I suggested might be due to the longer life outside the human body of this delicate and uncultivatable bacillus under such conditions, and to the number of insect bites in such climates affording points of entrance for the bacilli into the sub-epithelial layers of the skin, where they best flourish. Another important point is the much greater susceptibility of children and young adults to infection, as data of over 4,000 cases indicated that 50 per cent. of the infections occurred during the first two decades of life, and 75 per cent. in the first three ; the frequency of tuberculous infections of children being of interest in this connection.

*Prophylaxis.*—Hitherto we have been dependent on life-long segregation of lepers, which has produced a slow decline of the disease in Norway, Sweden, Iceland, Hawaii, and recently in Cyprus, Jamaica and British Guiana ; but is impracticable in most backward highly-infected tropical countries on account of its cost. Moreover, this policy is greatly handicapped by the inevitable hiding of the earlier stages of the disease as long as no effective treatment is known, resulting in the cases not being isolated until they have had numerous opportunities of infecting others of their household, thus maintaining the disease.

*Recent advances in Treatment.*—In leprosy there is a very nicely-balanced struggle between the bacilli and the invaded tissues, with a tendency, especially in the nerve form, for the infection to die out, but not before it has produced serious and crippling mutilations of the hands and feet. Numerous remedies, such as iodides, tuberculin, leprolin, nastin, etc., produced only temporary improvement, while the old Indian drug, chaulmoogra oil, retards the progress, but fails to clear up any but a few incipient cases in oral doses which can be tolerated. In 1913 Dr. Heiser[3] in the Philippines obtained 11 per cent. of apparent cures, with long series of painful intramuscular injections of the oil, which Indian patients would not tolerate.

In 1916, having previously obtained greater benefit from gynocardic acid* by the mouth, I made some sodium gynocardate, which, although painful, was more effective by injection. Further research showed that this preparation could be safely injected intravenously with even far better results, and in some cases it produced local softening and inflammation of the nodules with extensive breaking up of the bacilli in the body, followed by great improvement. However, sodium hydnocarpate, prepared from higher melting-point acids of chaulmoogra and hydnocarpate oils, proved still more effective, and a number of cases lost all signs of the disease and became uninfective owing to disappearance of the lepra bacilli.

Next I obtained similar results with sodium morrhuate

* The lower melting-point unsaturated fatty acids of chaulmoogra oil.

made from cod liver oil on the same lines, and with sodium soyate from soya bean oil,[4] thus establishing the important principle that effective preparations can be obtained by making soluble preparations of the unsaturated fatty acids of several oils, which have since been added to by Muir[5] and others.

A further important practical advance was made when Dean and Hollmann[6] in Hawaii showed that ethyl ester chaulmoograte prepared from chaulmoogra oil was effective by intramuscular injection, which is a more convenient mode of administration than giving the sodium salts intravenously, and is now in very general use in leprosy in many parts of the world, with results far superior to former methods of treatment.

To quote only the latest and most conclusive figures which have been just sent to me by Dr. Wade, and from the Cullion Leper Settlement in the Philippines of a trial in over 4,000 lepers, cases treated for 6 to 9 months showed improvement in 74 per cent., and those with over one year's treatment in no less than 93 per cent., while a number had completely cleared up.

*Prophylactic value of the Improved Treatment.*—In addition to the curative effects—for a few of my first cases have remained well for six to eight years—the fact that in many countries lepers are coming forward in the early stages of the disease in large numbers has enormously simplified the whole problem, for such early cases can be treated as out-patients in hospital clinics, as in Calcutta, and cleared up before they have infected their households, thus cutting off the sources of infection, and at last we are in a position to reduce the incidence of leprosy far more rapidly than hitherto.

*Mode of Action of the Drugs.*—Various explanations have been put forward to explain the essential action of these preparations in gradually destroying the acid-fast bacillus of leprosy, which have an obvious bearing on their use in tuberculosis, as proposed by me some six years ago. Thus, Walker and Sweeney[7] found that 1 in 100,000 strength of sodium hydnocarpate killed acid-fast bacilli in avian tubercle, and suggested a direct action on the lepra bacilli; but the fact that sodium morrhuate, with which they could get no action in vitro on acid-fast bacilli, is undoubtedly effective in leprosy, is much against this theory, although recent work of Campbell and Kieffer[8] appears to show some direct bactericidal effect of cod liver oil on tubercle cultures, so more research is required on this point. Shaw-Mackenzie[9] showed that sodium gynocardate and morrhuate doubled the digestive action of pancreatic lipase on fats, which led me to estimate the lipase in the sera of lepers kindly sent me by Dr. Muir, with the result[10] that untreated nodular cases showed very low lipase content, while it was far higher in treated cases, except one who had just had a severe reaction, during which the ferment may have been used up in dissolving the fatty coating of the lepra bacilli, and Muir has confirmed my observations, which afford a possible clue to action of these remedies.

*Sodium Morrhuate in Tuberculosis.*—In 1919[11, 12] I published the reports of several workers, who kindly tried injections of sodium morrhuate for me in tuberculous disease of various kinds, with promising results; and it has been found that given by the intravenous method, as in leprosy, it has a specific action on tuberculous lesions, but may be too active, as in the case of tuberculin, although beneficial results have been reported in South Africa, Brazil, etc. Dr. Boelke[13] of Sydney has just reported excellent results from a four years' trial of small subcutaneous doses of solutions freshly prepared on the days of injection, and he has shown that a local reaction, which may occur at the site of injection, very similar to one due to tuberculin, is specific for tuberculous disease, and also a sign that the dose should be slightly reduced, and this method appears to safeguard the use of the drug against severe and possibly harmful general reactions. If his results are confirmed by extensive trials now being carried out by several experts, it may prove possible to use sodium morrhuate with advantage in tuberculosis clinics. In cases of surgical tuberculosis, swelling up of enlarged glands or increased discharge from tuberculous sinuses is followed by decrease of the glands or the discharge, while in pulmonary cases decrease of fever, expectoration and in the number of the bacilli in the sputum are all favourable signs which may follow its use.

Thus the recent advances in the treatment of leprosy afford hope of being applicable, in some degree at any rate, to tuberculous disease due to closely allied acid-fast bacilli, and so are of general interest.

### REFERENCES.

[1] *Brit. M. J.* 1922, i. 987.
[2] *Indian J. M. Research,* 1923, xi. 239.
[3] *U.S. Pub. Health Rept.,* 1918, xxviii. 1,855.
[4] *Practitioner,* 1921, cvii. 77.
[5] *Indian J. M. Research,* 1923, xi. 543.
[6] *J. Cutan. Dis.,* 1919, xxxvii. 367.
[7] *J. Infect. Dis.,* 1920, xxvi. 238.
[8] *Am. Rev. Tuberculosis,* 1922, vi., No. 10.
[9] *Med. Press,* August 18th, 1920.
[10] *Brit. M. J.,* 1923, ii. 11.
[11] *Ibid.,* 1919, i. 147.
[12] *Indian J. M. Research,* 1919, vii., Spec. No., p. 236.
[13] *Brit. M. J.,* 1923, ii. 1,249.

